# Evaluation of [^11^C]UCB-A positron emission tomography in human brains

**DOI:** 10.1186/s13550-024-01117-2

**Published:** 2024-06-17

**Authors:** Mengfei Xiong, Mark Lubberink, Lieuwe Appel, Xiaotian Tsong Fang, Torsten Danfors, Eva Kumlien, Gunnar Antoni

**Affiliations:** 1 Molecular Imaging and Medical Physics, Department of Surgical Sciences, Uppsala University, Entrance 70, 75185 Uppsala, Sweden; 2 Department of Medical Sciences, Uppsala University, Uppsala, Sweden; 3 Department of Medicinal Chemistry, Uppsala University, Uppsala, Sweden; 4 Julius Clinical BV, Zeist, The Netherlands

**Keywords:** [^11^C]UCB-A, Kinetic modelling, PET, SV2A

## Abstract

**Background:**

In preclinical studies, the positron emission tomography (PET) imaging with [^11^C]UCB-A provided promising results for imaging synaptic vesicle protein 2A (SV2A) as a proxy for synaptic density. This paper reports the first-in-human [^11^C]UCB-A PET study to characterise its kinetics in healthy subjects and further evaluate SV2A-specific binding.

**Results:**

Twelve healthy subjects underwent 90-min baseline [^11^C]UCB-A scans with PET/MRI, with two subjects participating in an additional blocking scan with the same scanning procedure after a single dose of levetiracetam (1500 mg). Our results indicated abundant [^11^C]UCB-A brain uptake across all cortical regions, with slow elimination. Kinetic modelling of [^11^C]UCB-A PET using various compartment models suggested that the irreversible two-tissue compartment model best describes the kinetics of the radioactive tracer. Accordingly, the Patlak graphical analysis was used to simplify the analysis. The estimated SV2A occupancy determined by the Lassen plot was around 66%. Significant specific binding at baseline and comparable binding reduction as grey matter precludes the use of centrum semiovale as reference tissue.

**Conclusions:**

[^11^C]UCB-A PET imaging enables quantifying SV2A in vivo. However, its slow kinetics require a long scan duration, which is impractical with the short half-life of carbon-11. Consequently, the slow kinetics and complicated quantification methods may restrict its use in humans.

**Supplementary Information:**

The online version contains supplementary material available at 10.1186/s13550-024-01117-2.

## Background

The synaptic vesicle glycoprotein 2A (SV2A) is an integral membrane protein ubiquitously located in most glutamatergic and γ-aminobutyric acid (GABA)-ergic neuron terminals throughout the brain [1, 2]. In immunostaining, it is co-located with synaptophysin, a vesicle protein that served as a gold standard for quantifying synaptic density [3, 4]. Studies have revealed that SV2A has a crucial role in regulating neurotransmission and is vital in seizures and epilepsy development [5, 6]. Of particular relevance, SV2A has been identified as the molecular target of levetiracetam, an antiepileptic drug widely used in clinical practice [6]. This discovery not only provides insight into the mechanism of action of levetiracetam but also implies the diagnostic and therapeutic potential of targeting SV2A in the management of epilepsy and related disorders [7].

Based on the chemical structures of levetiracetam, UCB Pharma developed a series of derivatives with high affinities and specificities towards SV2A to estimate synaptic density in living subjects using positron emission tomography (PET) imaging [8]. Among all the candidates, one imidazole derivative and two compounds with lutidine substructures stood out. The former compound was named [^11^C]UCB-A, while the latter two were [^11^C]UCB-J and [^18^F]UCB-H [8].

The three compounds underwent initial animal evaluations before [^11^C]UCB-J and [^18^F]UCB-H progressed to human studies [4, 9–11]. In rodents, all three radioactive tracers displayed high uptake in grey matter areas [11–13]. In comparison to [^11^C]UCB-J and [^18^F]UCB-H, [^11^C]UCB-A exhibited slightly slower kinetics. The slow kinetics were confirmed in comparison with other SV2A tracers in non-human primates [14]. Further PET scans with blocking agents were performed in rats and pigs [11]. The results revealed that [^11^C]UCB-A displayed a dose-dependent blocking effect and a rapid and complete displacement after levetiracetam administration in pigs [11]. These findings suggested the potential utility of [^11^C]UCB-A for measuring SV2A in humans, warranting further exploration of its efficacy in human studies [11–13].

Validation of [^11^C]UCB-J and [^18^F]UCB-H PET demonstrated reversible kinetics in humans [4, 15]. Because [^18^F]UCB-H has a relatively low affinity and signal-to-noise ratio in PET images [8, 15], [^11^C]UCB-J became the most widely investigated PET tracer for SV2A in humans, for its relatively higher affinities and optimal kinetics [4]. Since then, [^11^C]UCB-J PET has been explored to identify synaptic changes that occur in many neurodegenerative diseases and psychiatric disorders [16–18]. In Alzheimer’s disease, [^11^C]UCB-J binding in the hippocampus decreased by 41% compared with cognitively normal participants [17]. In patients with medial temporal lobe epilepsy, the lower [^11^C]UCB-J PET signals corresponded to the ipsilateral epileptogenic lobe, which confirms the opportunity for SV2A PET and encourages further investigation in synaptic imaging [16].

In this study, we conduct a first-in-human [^11^C]UCB-A study in healthy subjects using PET imaging. The current study aims to characterise the binding properties and pharmacokinetics of [^11^C]UCB-A and evaluate the SV2A-specific binding. For this purpose, we first employ kinetic modelling on [^11^C]UCB-A PET data using tracer kinetic models. To investigate if [^11^C]UCB-A uptake is dependent on perfusion, we examine the relationship between [^11^C]UCB-A uptake and cerebral blood flow using data from a [^15^O]water scan. Second, we assess the specific binding of [^11^C]UCB-A to SV2A in subjects with an additional blocking scan after pre-treatment of levetiracetam.

## Methods

### Subjects

Twelve healthy subjects (7 M/5F; age: 37.3 ± 12.9 years, range: 22–61 years) were recruited through the assistance of Clinical Trials Consultants AB (Uppsala, Sweden). Potential participants received written and oral information about the study and were interviewed about their health and lifestyle habits. No subject had a record of taking levetiracetam or other anti-SV2A drug.

### Radiochemistry

The radiosynthesis of [^11^C]UCB-A was conducted onsite using a two-step synthesis route. The precursor was reacted with [^11^C]methyl triflate and trifluoroacetic acid, as previously described [11]. The precursor for synthesising [^11^C]UCB-A was provided by UCB Pharma (Braine-l’Alleud, Belgium). [^11^C]UCB-A was obtained with a radiochemical purity higher than 98%, and the molar activity was 33–93 GBq/µmol (n = 14). The production followed Good Manufacturing Practice and was in accordance with the general chapter “Radiopharmaceuticals” in the European Pharmacopoeia.

### Study design and PET/MR imaging

All subjects underwent baseline PET scans with [^15^O]water, followed by [^11^C]UCB-A scans and structural 3-dimensional T1-weighted magnetic resonance imaging (MRI). Prior to the PET/MRI examination, subjects fasted for a minimum of 4 h and were restrained from alcohol, coffee, and nicotine products for at least 12 h. Two subjects (male; age: 22–23 years) participated in an additional blocking experiment. After baseline scans, the subjects got a break and a light lunch before undergoing blocking scans, following the same scanning procedures as in the first step. A single oral dose of 1500 mg (15.5 and 18.5 mg/kg) levetiracetam (Keppra®, UCB Pharma SA, Belgium) was administered 100 min before the start of the second [^11^C]UCB-A scan. The interval between the baseline and blocking [^11^C]UCB-A scans was 4 h, with subjects fasting for 2 h before the second [^11^C]UCB-A scan.

PET scans were acquired on a SIGNA PET/MR scanner (GE Healthcare, Waukesha). Subjects were positioned supine in the scanner and first received an automated bolus injection of [^15^O]water (5 MBq/kg) and underwent a 6-min list mode PET scan starting simultaneously with the injection, followed by a 90-min list mode PET scan starting simultaneously with bolus injection of 6 MBq/kg (432 ± 56 MBq) [^11^C]UCB-A (10 mL at 0.8 mL/s followed by 30 mL saline at 2 mL/s). The time between the start of the two PET scans was at least 15 min to allow for the decay of the remaining [^15^O]water.

### Data acquisition and reconstruction

[^15^O]water PET data were reconstructed into 22 frames (1 × 10, 8 × 5, 4 × 10, 2 × 15, 3 × 20, 2 × 30, 2 × 60 s) using an ordered subset expectation maximisation (OSEM) algorithm with 4 iterations and 28 subsets, including time-of-flight (TOF) and point spread function (PSF) recovery and 5 mm Gaussian post-filter. The reconstructed matrix size was 128 × 128 × 89 voxels with a voxel size of 2.34 ×  × 2.34 × 2.78 mm) [19]. [^11^C]UCB-A PET data was reconstructed into 25 frames (6 × 10, 3 × 20, 2 × 30, 2 × 60, 2 × 150, 4 × 300, and 6 × 600 s) using TOF-OSEM with PSF and 3 mm Gaussian post-filter. Attenuation correction was based on a zero-echo time (ZTE) MR scan acquired before the PET scans using previously described procedures [20, 21].

### Metabolite analysis

Blood was taken continuously (3 mL/min) through a radial artery cannula during the entire [^15^O]water scan and the first 10 min of the [^11^C]UCB-A scan. Radioactivity was measured by an online detector next to the patient’s wrist (Twilite Two, Swisstrace, Switzerland). For [^11^C]UCB-A PET, additional blood samples were taken at 5, 10, 20, 30, 45, 60, 75, and 90 min post tracer injection (p.i.) for measuring radioactivity in whole blood and plasma, as well as for metabolite analysis.

Intact fraction and metabolites of [^11^C]UCB-A in plasma were determined via analytical reversed-phase high-performance liquid chromatography (HPLC), following methods updated from published protocols [11]. Plasma fractions were obtained from blood samples through centrifugation. Duplicate plasma samples (0.6 mL each) were transferred to new tubes, where an equal volume of acetonitrile was added. The mixture was then centrifuged at 13,200 rpm at 4 °C for 1 min, and the resulting supernatant was filtered under the same conditions. For HPLC sample preparation, 1 mL of filtered plasma was diluted with 1 mL of water and supplemented with 20 µL of 1 mg/mL UCB-A. The resulting 1.8 mL mixture was injected into the HPLC column (ASPEC® Gilson, Middleton, USA), utilising an elution buffer consisting of 8.1 mM ammonium carbonate (pH 8.8) and methanol (45:55, v:v) as the mobile phase. The radioactive signal was detected at 260 nm, and three fractions were collected and quantified using a scintillation counter.

### Image analysis

Frame-by-frame motion correction was applied to PET images using a summed image of the first 3 min of the scan as a reference. No excessive motion or rotation was seen in the subjects (> 5 mm or > 2 degrees in any direction).

Regional distribution of [^15^O]water and [^11^C]UCB-A was analysed using kinetic models based on a metabolite-corrected plasma input function. To generate the plasma input function of [^11^C]UCB-A, the blood curve from the online sampler was corrected for delay using a single tissue compartment model to fit the total true count rate curves, including a delay parameter. A single exponential was then fitted to the sampler curve between 3 and 7 min p.i., and the sampler curve was calibrated by scaling the value of this fit at 5 min p.i. to match the whole blood activity in the blood sample as measured with a well counter that was cross-calibrated with the PET scanner. The sampler curve was extrapolated to the entire scan length by a multi-exponential fit to the whole-blood activity measured in the subsequent blood samples. Then, a plasma time-activity curve (TAC) was computed by multiplication of the whole-blood TAC by a linear fit to the plasma/whole-blood ratio over time and subsequently multiplicated with a sigmoid fit to the parent fraction data, resulting in a metabolite-corrected plasma input curve. The calibration factor was obtained from [^15^O]water scan and used also in [^11^C]UCB-A. External dispersion was ignored as the distance between the arterial cannula and detector was less than 5 cm.

To generate TACs in various regions of interest (ROI), summed dynamic PET images (0–5 min) were co-registered with the corresponding MRI and segmented in PVElab using SPM8 (Wellcome Department of Cognitive Neurology, London, UK) [22, 23]. Individual MR images were resliced to the PET matrix size and applied to the Hammers atlas [24]. Regional TACs in each ROI from the atlas were then extracted without partial volume effect correction. The grey matter (GM) regions in the cortical areas were selected and merged into 5 large ROIs: frontal (Front), temporal (Temp), medial temporal (Med Temp), parietal (Pari), and occipital lobes (Occi). For [^11^C]UCB-A scans, centrum semiovale (CS) was automatically defined using a 98% threshold in the SPM8 white matter images after smoothing them with a 7 mm Gaussian filter, including only voxels in the upper half of the axial field of view to avoid including voxels in pons.

[^11^C]UCB-A data was analysed using four single- and two-tissue compartment models with reversible (1T2k, 2T4k) and irreversible tracer binding (1T1k, 2T3k) and a fitted blood volume parameter [25]. Regional distribution volumes (V_T_) were estimated by reversible compartment models. Non-displaceable binding potential BP_ND_ was obtained directly from the 2T4k model, while the 2T3k model yielded the net influx rate K_i_ (mL/cm^3^/min). Fits with standard errors of macro parameters (K_i_, V_T_) over 50% were discarded. The Akaike Information Criterion (AIC) was used to select the optimal kinetic model [26]. Graphical Logan and Patlak analyses were also performed using both plasma input and reference region [27, 28]. Centrum semiovale was proposed as a reference region for simplified analysis for [^11^C]UCB-J as white matter lacks SV2A [4, 29, 30]. Thus, we explored the possibility of simplified quantification using centrum semiovale as the reference region in graphical analyses for [^11^C]UCB-A. Time intervals of 10, 20, 30, and 60 to 90 min, as well as 30–70 min, were explored [31, 32].

Based on the ROI-based analysis results, parametric images of K_i_ were calculated using the graphical methods for [^11^C]UCB-J PET. Each subject’s PVElab ROI template was transferred to the corresponding parametric binding parameter image, and parametric K_i_ values for the five studied brain regions were compared to those computed using ROI-based analysis.

For [^15^O]water scan data, cerebral blood flow (CBF) was determined directly from uptake rate K_1_ (mL/cm^3^/min), computed via non-linear regression of the operational equation of the single tissue compartment model with a fitted delay [19]. The blood volume (V_B_) was set to 5%.

SV2A-specific binding was determined using data from baseline and blocking scans in two subjects. Based on the results of the optimal model for describing [^11^C]UCB-A kinetics, the estimated SV2A occupancy was determined by either regional K_i_ or V_T_ values from plasma Patlak and Logan models as the relative change of [^11^C]UCB-A specific binding between baseline and blocking scans in all 5 ROIs using Lassen plots [29, 33–35].

### Statistical analysis

Statistical analyses were conducted using GraphPad Prism 10.2.1 (GraphPad Software, Boston, Massachusetts, USA). The normality of all datasets was confirmed by the Shapiro–Wilk test and the Kolmogorov–Smirnov test. Simple linear regression (R^2^) and Pearson correlation (r) analyses were performed to determine the relationships between parameters. The area under the curve (AUC) was calculated for SUV TACs. AUCs in all 5 ROIs and centrum semiovale were compared in a two-way analysis of variance (ANOVA) with Tukey’s multiple comparisons using brain region and subject as variables. Confidence intervals in all analyses were set to 95%. Data are reported as mean ± standard deviation (SD) if not otherwise stated.

## Results

### Kinetic modelling of [^11^C]UCB-A

**Fig. 1:**
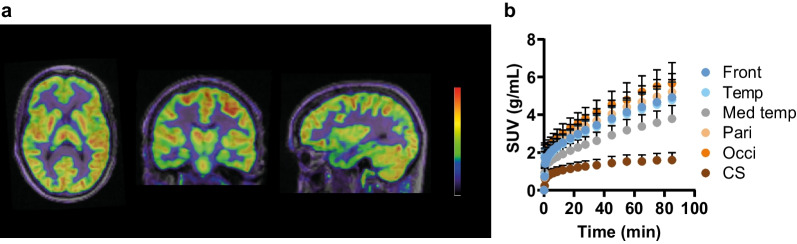
**a** An [^11^C]UCB-A PET image (averaged 0–90 min), expressed in standardised uptake values (SUV), in a healthy volunteer. The PET image is fused with the MR image from the subject. **b** Mean time-activity curves (TACs) based on SUV in the frontal lobe (Front), temporal lobe (Temp), medial temporal lobe (Med temp), parietal lobe (Pari), occipital lobe (Occi), and centrum semiovale (CS) from all subjects

Retention of [^11^C]UCB-A in brain grey matter was prominent and relatively homogeneous (Fig. 1a). The occipital lobe exhibited the highest uptake, followed by the parietal, frontal, temporal, and medial temporal lobes (Fig. 1). White matter regions, such as the centrum semiovale, displayed less [^11^C]UCB-A uptake, significantly lower than that observed in grey matter (*p* < 0.0001). The kinetics of [^11^C]UCB-A within the brain were slow. Over the 90-min scan duration, uptake in grey matter regions continued to rise without reaching a peak, suggesting continuous tracer delivery and binding (Fig. 1b).

**Fig. 2:**
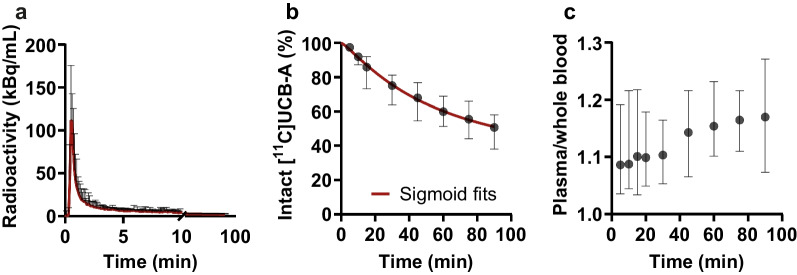
**a** Plasma input, **b** intact fraction of [^11^C]UCB-A with Hill sigmoid fits during 90 min of baseline scans and **c** plasma-to-whole blood ratio. Data points are shown as mean ± SD

Blood clearance of [^11^C]UCB-A was slow, with whole blood concentrations remaining relatively constant after 15 min (Fig. 2a). The intact [^11^C]UCB-A fraction in plasma was 86% at 15 min and 51% at 90 min, indicating high metabolic stability and limited excretion of the tracer (Fig. 2b). Plasma-to-whole blood ratio slowly increased from 1.09 to 1.17 during the scan (Fig. 2c).

**Fig. 3:**
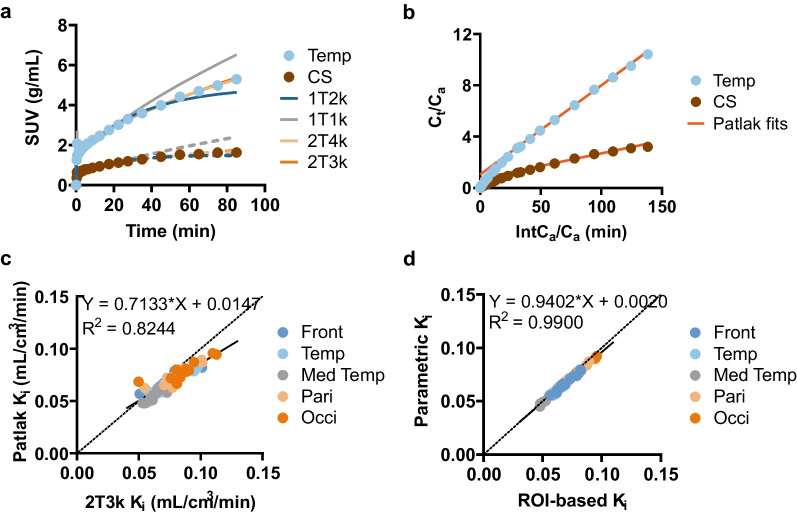
**a** Example of TACs in the temporal lobe (Temp) and centrum semiovale (CS) and fits with single- and two-tissue compartment models assuming reversible (1T2k; 2T4k) and irreversible (1T1k; 2T3k) binding. **b** Plasma-input Patlak plots in the same region of the subject. **c** Correlation between K_i_ estimated from graphical Patlak analysis (30–70 min) and irreversible two-tissue compartment model (2T3k) for 5 cortical regions as in (**d**) across all subjects. **d** Simple linear regression analysis (R^2^) between K_i_ estimated from region-based (ROI) and voxel-based (parametric) analyses (*p* < 0.0001)

Of the four evaluated compartmental models, both 1TC models provided poor fits of the data. In contrast, the 2TC models fitted the data well (Fig. 3a). According to the Akaike criteria, the reversible 2T4k model provided the best fits in 35 out of 60 (12 subjects × 5 ROIs) fits, whereas the irreversible 2T3k model was preferred in 25 out of 60 fits. On the other hand, the 2T4k model often resulted in unreliable V_T_ and BP_ND_ estimations with large standard errors (> 50%) in all ROIs, whereas no K_i_ values had standard errors over 25%. Thus, the irreversible 2T3k model that provided robust K_i_ estimates was the preferred compartmental model for quantifying [^11^C]UCB-A kinetics in humans. The detailed overview of regional macro parameters of the 2T3k model is displayed in Table 1.

**Table 1:** Regional kinetic parameters K_1_ and K_i_ of [^11^C]UCB-A estimated by two-tissue irreversible (2T3k) kinetic models (mean ± SD)

Brain regions	K_1_ (mL/cm^3^/min)	K_i_ (mL/cm^3^/min)
Frontal	0.132 ± 0.028	0.077 ± 0.013
Temporal	0.128 ± 0.026	0.075 ± 0.011
Medial Temporal	0.104 ± 0.018	0.063 ± 0.008
Parietal	0.139 ± 0.029	0.081 ± 0.013
Occipital	0.153 ± 0.032	0.087 ± 0.016
Centrum semiovale	0.054 ± 0.011	0.020 ± 0.003

Plasma-input Patlak analysis consistently displayed a linear phase between 10 and 70 min of the scan but often showed a slight downward trend after 70 min (Fig. 3b). Linear regression analyses revealed that K_i_ derived from Patlak analysis using data between 30 and 70 min agreed best with the 2T3k model, albeit with a slight proportional underestimation in Patlak K_i_ (Fig. 3c). Consequently, Patlak K_i_ generated from the 30–70 min data interval was selected for further studies. Moreover, parametric Patlak K_i_ was in excellent agreement with ROI-based analysis (*p* < 0.0001, Fig. 3d).

### Cerebral blood flow

**Fig. 4:**
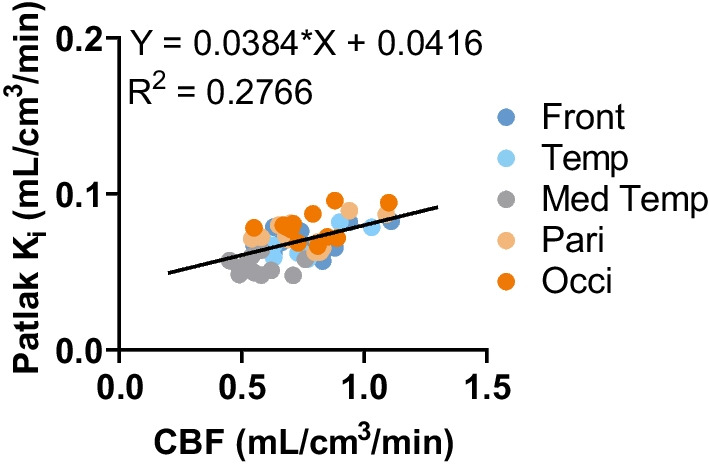
Simple linear regression analysis between cerebral blood flow (CBF) and Patlak K_i_

The mean CBF in the five studied brain lobes across subjects is presented in Additional file 1: Table 1. In total brain grey matter, the mean CBF was 0.57 ± 0.13 mL/cm^3^/min. To explore the potential influence of perfusion on K_i_, we conducted a correlation analysis between Patlak K_i_ and CBF. The modest but significant correlation (*p* < 0.0001) indicated that the variation in CBF can partly explain the variability observed in [^11^C]UCB-A K_i_ values (Fig. 4).

### SV2A blocking experiment

**Fig. 5:**
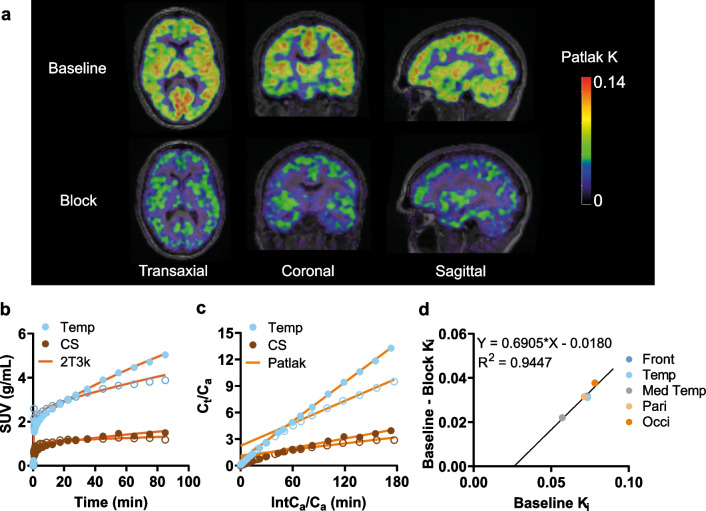
**a** Parametric K_i_ images in a healthy subject at baseline and blocking scan. The subject received 1500 mg of levetiracetam before the blocking scan. **b** TACs in the temporal lobe (Temp) and centrum semiovale (CS) obtained with an irreversible two-tissue compartment model (2T3k). **c** Graphical Patlak plots for the same regions as in (**b**). Closed symbols represent the baseline scan, and open symbols represent the blocking scan. **d** Lassen plot using Patlak K_i_ (30–70 min) from five grey matter areas at baseline and blocking scans. Front: frontal lobe; Med temp: medial temporal lobe; Pari: parietal lobe; Occi: occipital lobe

Figure 5a shows parametric K_i_ images in one healthy subject before and after receiving levetiracetam. Brain TACs of baseline and blocking scans in grey matter started to separate after 30 min. At the same time, only minor differences were seen in the centrum semiovale until the end of the scan (Fig. 5a, b). In data from blocking scans, the fits overestimated the measured TACs after 60 min (Fig. 5b). This indicated that [^11^C]UCB-A and SV2A bind reversibly, which becomes more evident after blocking.

Further, K_i_ at baseline and blocking conditions in grey matter ROIs and centrum semiovale were determined by Patlak analysis (Table 2). The example data from subject 2 with Patlak fits is shown in Fig. 5c. The reduction of K_i_ values between the two scans was used to generate Lassen plots to estimate occupancy (Fig. 5d). Comparing baseline and blocking scans, mean Patlak K_i_ in grey matter decreased from 0.063 to 0.045 mL/cm^3^/min (28%) and from 0.071 to 0.040 mL/cm^3^/min (43%) in the two subjects, with occupancy values of 64 and 69%, respectively. In comparison, K_i_ in centrum semiovale decreased from 0.014 to 0.012 (16%) and from 0.018 to 0.012 mL/cm^3^/min (36%). The reduction observed in the centrum semiovale is more than fifty percent of the reduction observed in the grey matter, indicating a considerable amount of specific binding in the centrum semiovale.

**Table 2:** K_i_ values using graphical Patlak analysis (30–70 min) in the studied brain regions at baseline and after blocking with levetiracetam

Brain regions	Subject 1			Subject 2		
	K_i_ (mL/cm^3^/min)		Decrease (%)	K_i_ (mL/cm^3^/min)		Decrease (%)
	Baseline	Block		Baseline	Block	
Frontal	0.065	0.045	31	0.073	0.041	43
Temporal	0.062	0.046	26	0.073	0.042	42
Medial temporal	0.048	0.039	19	0.057	0.035	38
Parietal	0.065	0.046	30	0.071	0.040	44
Occipital	0.072	0.047	34	0.078	0.040	48
Centrum semiovale	0.014	0.012	16	0.018	0.012	36

## Discussion

In this study, for the first time, we characterised the bindings of [^11^C]UCB-A in humans using kinetic modelling. [^11^C]UCB-A showed a high uptake in grey matter, which was consistent with the distribution patterns observed for [^11^C]UCB-J and [^18^F]UCB-H [4, 11, 15]. The occipital lobe exhibited the highest net influx rate (K_i_) for [^11^C]UCB-A, contrasting with [^11^C]UCB-J, for which the temporal cortical regions exhibited the highest uptake [4]. No data regarding binding in the occipital cortex is available for [^18^F]UCB-H [15, 36]. Across all three tracers, the medial temporal region consistently exhibited the lowest tracer uptake. In the white matter, such as centrum semiovale, directly comparing binding among the three tracers was not feasible due to diverse outcome parameters generated by different kinetic models. Therefore, we compared relative ratios of binding in the centrum semiovale to the grey matter. For [^11^C]UCB-A, K_i_ in centrum semiovale was 26% of that in grey matter, similar to [^11^C]UCB-J for which V_T_ (2T4k) in centrum semiovale was 29% of that in the grey matter, but lower than [^18^F]UCB-H, for which V_T_ (Logan) in the centrum semiovale was 55% of that in grey matter [9, 29].

We applied full kinetic modelling and evaluated the most suitable model to characterise the kinetics of [^11^C]UCB-A. The irreversible 2T3k model was preferred to describe [^11^C]UCB-A kinetics. Consequently, the plasma-input Patlak plot was considered the most appropriate graphical analysis approach. These are in line with previous findings from preclinical [^11^C]UCB-A PET studies in pigs [11]. Slow uptake of [^11^C]UCB-A was seen during the baseline scans, suggesting apparent irreversibility when TACs of 90 min did not reach a peak.

Reversible binding of [^11^C]UCB-A was seen in displacement studies in rats and blocking studies in pigs with good fits using the 2T4k model and complete blocking of tracer binding after receiving levetiracetam [11]. However, a better fit with the 2T4k model was not achieved in blocking scans in the current study. Notably, the small downward curvature of data compared to model fits in the last 20 min of the scans (Figs. 3b and 5c) indicated that [^11^C]UCB-A binds reversibly to SV2A, but it would take much longer than 90 min to obtain robust V_T_ and BP_ND_ values. Accordingly, only 30–70 min of data were used in the plasma input-based Patlak analysis.

The apparent slow kinetics of [^11^C]UCB-A, which did not reach equilibrium during the PET scan, could be attributed to a combination effect of slow brain entry, slow plasma clearance, and slow target dissociation. The imidazole ring in the [^11^C]UCB-A structure was suspected of conferring highly hydrophobic and metabolically stable properties to [^11^C]UCB-A, which hampers its entry into the brain, leading to its retention in the plasma instead [8, 14]. For instance, in the temporal lobe, the K_1_ of [^11^C]UCB-A (0.128 ± 0.026 mL/cm^3^/min) was much smaller than for [^11^C]UCB-J (0.32 ± 0.04 mL/cm^3^/min) [4], supporting the explanation of slower brain entry for [^11^C]UCB-A compared to [^11^C]UCB-J.

Although the current study did not measure the free fraction in plasma (f_p_), the higher f_p_ of [^11^C]UCB-A (75%) compared to [^11^C]UCB-J (46%) in rhesus monkeys supported the assumption of slow plasma clearance of [^11^C]UCB-A [14]. Furthermore, our study found that the 50% intact fraction of [^11^C]UCB-A at 90 min indicated that the tracer was more stable than [^11^C]UCB-J and [^18^F]UCB-H, for which the parent fraction was around 20% at the same time [4, 15]. Additionally, dissociation of [^11^C]UCB-A from SV2A was exceptionally slow, as indicated by the extremely small dissociation rate constant k_4_ derived from the 2T4k model.

The specific binding of [^11^C]UCB-A towards SV2A was assessed by the net uptake rate (K_i_) differences between the two scans before and after blocking with levetiracetam. The estimated occupancies (64% and 69%) indicated a moderate to high level of binding affinity between [^11^C]UCB-A and SV2A. The occupancy values were below the maximum of 80% of occupancy estimated by [^11^C]UCB-J for the same levetiracetam dose [37], suggesting [^11^C]UCB-A displayed a less effective interaction with SV2A compared to [^11^C]UCB-J.

Centrum semiovale has been proposed as a reference region for simplifying analysis for [^11^C]UCB-J, given the minimal V_T_ in this region and the good agreement between V_T_ in centrum semiovale and V_ND_ in grey matter [4, 30]. We evaluated if centrum semiovale can be used as a reference region for [^11^C]UCB-A analysis using data from the SV2A blocking experiment. Blocking with levetiracetam substantially decreased K_i_ in the centrum semiovale, although to a lesser extent than in grey matter. This has also been observed in [^11^C]UCB-J: post-drug V_T_ measured by the 1T2k model decreased by 7% in the centrum semiovale and approximately 50% in the grey matter [29]. The decrease in binding observed in the centrum semiovale following blocking was evidently greater for [^11^C]UCB-A and was substantial compared to the reduction in the grey matter. The reduction in centrum semiovale has been suggested to be due to spill-over from grey matter or incomplete convergence of the OSEM algorithm [30]. We did re-do the analysis with block-sequential regularised expectation maximisation (BSREM) reconstructions, resulting in higher spatial resolution and hence less spill-over, but this did not diminish the large reduction in K_i_ in centrum semiovale (data not shown). Hence, centrum semiovale is not a suitable reference region for simplified [^11^C]UCB-A quantification.

Our study has several limitations: (1) The 90-min acquisition time for the [^11^C]UCB-A PET scan may not have been sufficient to reach equilibrium. Extending the scan duration to 120 min or longer would allow for capturing the tracer’s elimination phase, providing more comprehensive data. (2) The occupancy determined in the blocking study was conducted on only two subjects, which limited the statistical power of our findings. Nevertheless, the blocking effects in grey matter areas and the centrum semiovale were evident and consistent with findings reported for other SV2A tracers in the literature.

## Conclusions

The apparent irreversible kinetics and the necessity of arterial blood sampling and metabolite analysis for [^11^C]UCB-A quantification restrict the broad and simple use of [^11^C]UCB-A. A radioactive tracer with reversible kinetics would be preferable for quantitative readouts. However, despite these limitations, [^11^C]UCB-A shows high specificity, abundant brain distribution, and low inter-subject variability of the K_i_ values for all ROIs. Combined with a high blocking effect, [^11^C]UCB-A has the potential to compare SV2A expression in diseased and healthy conditions. It should be noted that the tracer binding is partially dependent on perfusion. This needs to be considered in future studies when enrolling patients with altered cerebral blood flow.

### Supplementary Information

Additional file 1.

## Data Availability

The data analysed in the current study are available in GraphPad table format and are available from the corresponding author upon reasonable request. Supplemental data can be found in the Excel document.

## Abbreviations

1T1k: Irreversible one-tissue compartment model
1T2k: Reversible one-tissue compartment model
2T3k: Irreversible two-tissue compartment model
2T4k: Reversible two-tissue compartment model
AIC: Akaike Information Criterion
AUC: Area under the curve
BP_ND_: Non-displaceable binding potential
CBF: Cerebral blood flow
HPLC: High-performance liquid chromatography
K_i_: Net influx rate, mL/cm^3^/min
MRI: Magnetic resonance imaging
OSEM: Ordered subset expectation maximisation
PET: Positron emission tomography
PSF: Point spread function
ROI: Region of interest
SUV: Standardised uptake value
SV2A: Synaptic vesicle glycoprotein 2A
TAC: Time-activity curve
V_ND_: Distribution volume of non-displaceable compartment
V_T_: Volume of distribution
